# P08 An usual case of pandrug-resistant *Serratia marcescens* in a 63 year-old-male: a case report

**DOI:** 10.1093/jacamr/dlad143.012

**Published:** 2024-01-03

**Authors:** Muhammad Shoaib Irshad, Wahaj, Muhammad Junaid Irshad

**Affiliations:** Maidstone and Tunbridge Wells NHS Trust; Abbottabad International Medical Institute; University Hospital Crosshouse

## Abstract

**Objectives:**

To highlight the growing burden of antimicrobial resistance in developing countries where there are no officially agreed local antibiotics guidelines. We are characterizing a case of growth of pandrug-resistant (PDR) *Serratia marcescens* in the urine culture of a 63-year-old-male.

**Patient and methods:**

Our patient, who had a history of recurrent renal stones, underwent multiple urological procedures (including ureteroscopy and DJ stenting) in various regional hospitals in northern Pakistan. It is postulated that he became infected during these repeated procedures which were done in various hospitals without adequate sterile equipment. He presented with recurrent UTIs to GPs and was subsequently treated with oral fosfomycin for over 1 year in total. His baseline renal function remains unknown due to the absence of online records, but he was noted to have CKD IV. Subsequently, during his later visits to the hospital, he developed sepsis and his urine cultures confirmed carbapenem-resistant *Pseudomonas aeruginosa*, which was treated with IV antibiotics as per the susceptibility report. He was prescribed IV piperacillin/tazobactam (Tazocin) for a total period of 3 months on various occasions due to repeated presentations with urosepsis. He improved clinically with IV piperacillin/tazobactam. Following this, the patient was lost to follow-up. The patient once again presented acutely unwell with urosepsis. This time urine cultures confirmed the presence of *S. marcescens* (May 2023). The patient had pandrug resistance to all antibiotics including IV piperacillin/tazobactam. However it was decided to treat him with IV piperacillin/tazobactam due to previous good results. The patient improved clinically but his renal functions declined to CKD stage V. He was once again lost to follow up. He presented again to renal consultants with uraemic symptoms and at the moment he is being counselled for dialysis due to his end-stage renal disease. At present the patient is having asymptomatic bacteriuria; his urine cultures remain positive without any symptoms. A more recent urine culture (September 2023) showed growth of *Escherichia coli* (ESBL). Due to lack of symptoms, renal consultants agreed to manage the patient without further antibiotics.

**Results:**

In May 2023, an antibiotic susceptibility profile was done under supervision of microbiologists. Unfortunately for the patient, the urine culture reported growth of *S. marcescens* and that the patient had pandrug-resistance. The profile included testing for the following antibiotics: amikacin, co-amoxiclav, aztreonam, cefepime, cefoxitin, ceftazidime/avibactam, cefalexin, co-trimoxazole, fosfomycin, meropenem, amoxicillin, ampicillin, cefaclor, cefixime, cefpodoxime, cefuroxime, ciprofloxacin, colistin, imipenem, nitrofurantoin, norfloxacin, polymyxin, piperacillin/tazobactam and sulbactam/cefoperazone. In September 2023, the second urine culture report with the growth of *E. coli* showed susceptibility to amikacin, cefoperazone/sulbactam, fosfomycin, imipenem and tigecycline. This report also confirmed resistance co-amoxiclav, ampicillin, cefixime, cefotaxime, ceftazidime, ceftriaxone, cefuroxime, cefradine, levofloxacin, nalidixic acid, nitrofurantoin, piperacillin/tazobactam and trimethoprim/sulfamethoxazole.

**Conclusions:**

Unfortunately, the urine culture and susceptibility report signify the threat this patient faces in case he develops further urine infections. It is imperative to encourage development of local guidelines in developing countries too. We would recommend a multidisciplinary approach in treating difficult and long-term infections with constant support of microbiologists.

